# Supplementary material for: Targeting tumor–stromal interactions in triple-negative breast cancer using a human vascularized micro-tumor model
Source: Breast Cancer Res. 2024 Jan 5;26:5. doi: 10.1186/s13058-023-01760-y (PMC10768273; doi:10.1186/s13058-023-01760-y)
Supplement: Supplementary file 1 — Additional file 1. Supplemental figures. [file 13058_2023_1760_MOESM1_ESM.pdf]

Appendix A Supplementary materials

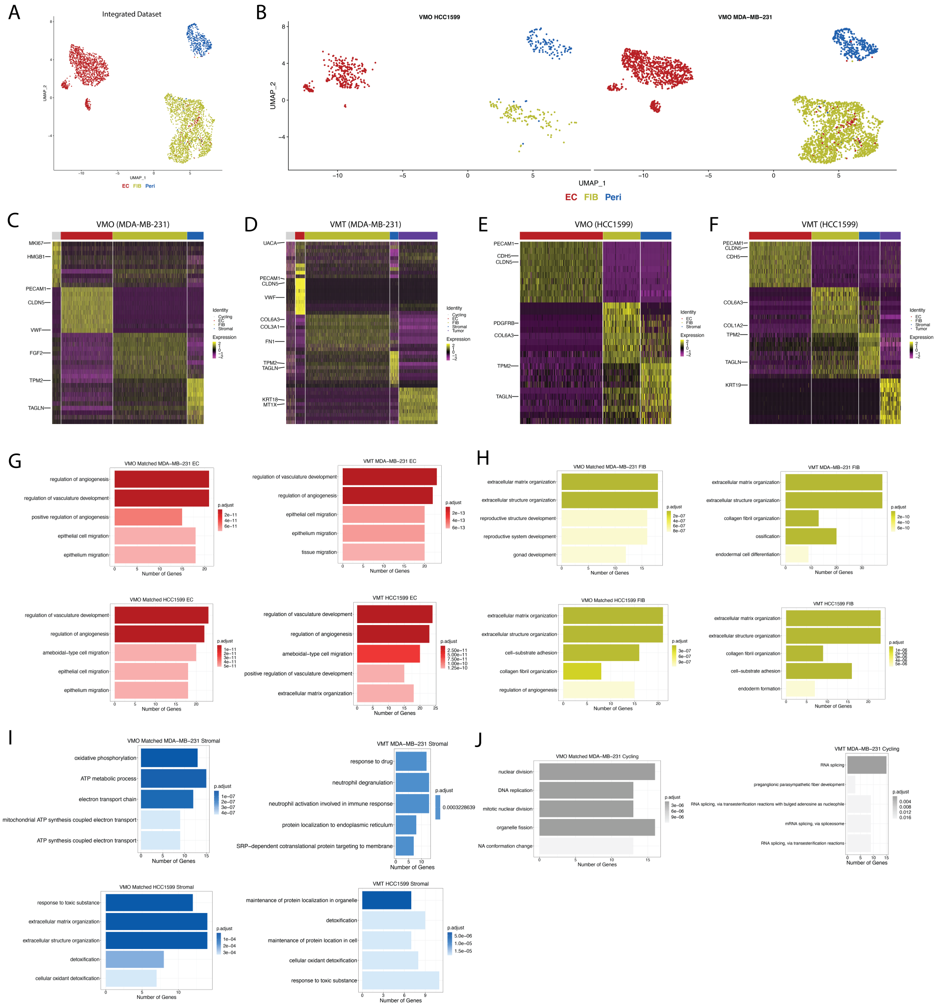

Fig. A1: **Differential gene expression analyses of individual datasets reveal distinct pathway activation dependent on cell type in both VMTs and VMOs.** **(A)** UMAP showing integrated VMO dataset, **(B)** shown with datasets split by VMO matched to HCC1599 and MDA-MB-231 VMT, respectively. **(C)** Heatmap showing top 10 DEGs for VMO matched to MDA-MB-231 VMT. **(D)** Heatmap showing top 10 DEGs for MDA-MB-231 VMT. **(E)** Heatmap showing top 10 DEGs for VMO matched to HCC1599 VMT. **(F)** Heatmap showing top 10 DEGs for HCC1599 VMT. **(G)** Gene Ontology Biological Processes pathway analyses for endothelial cells (EC) in each dataset. **(H)** Gene Ontology Biological Processes pathway analyses for fibroblasts in each dataset. **(I)** Gene Ontology Biological Processes pathway analyses for pericytes/stromal cells in each dataset. **(J)** Gene Ontology Biological Processes pathway analyses for cycling cell population in each dataset.

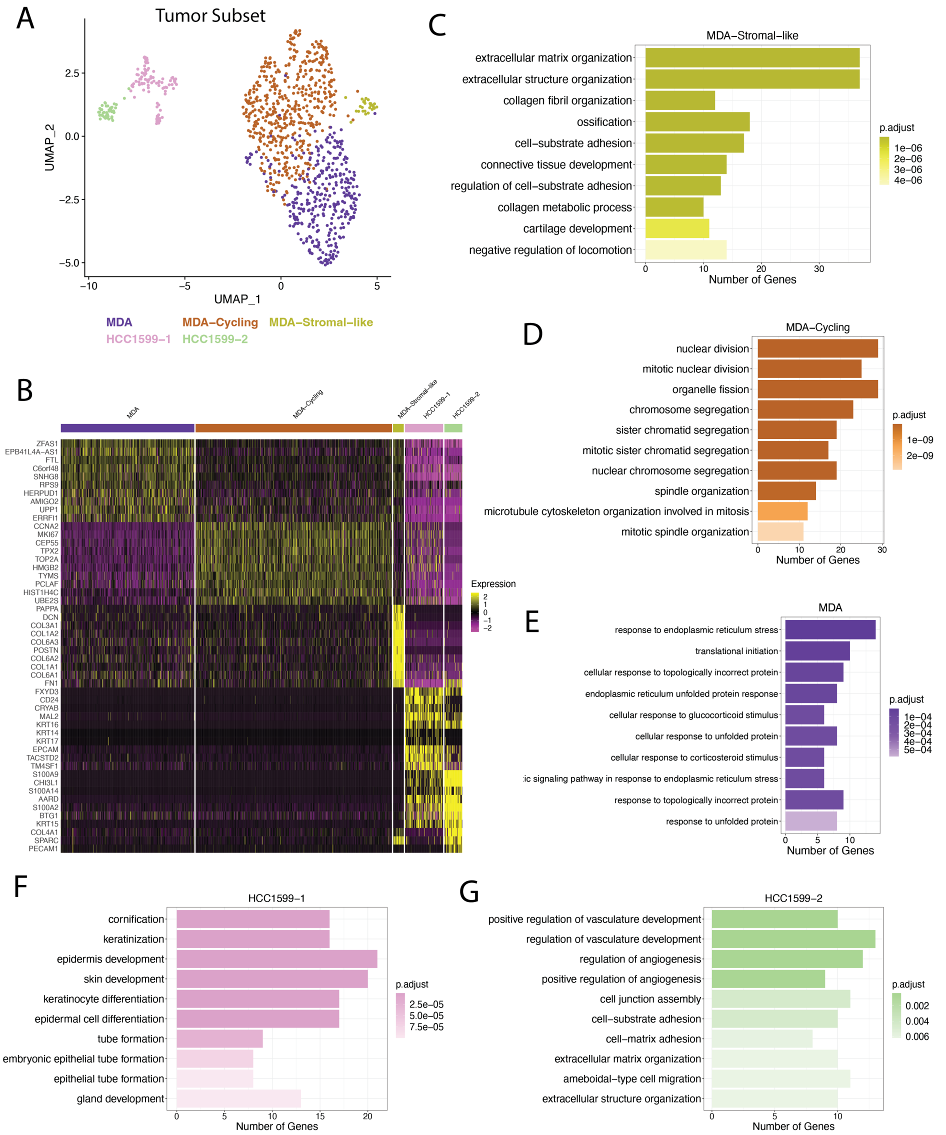

Fig. A2: **Tumor subset from integrated VMT dataset highlights differences in TNBC cell populations. (A)** UMAP showing cell clusters for integrated VMT dataset. **(B)** Heatmap showing top 10 DEGs for tumor populations from MDA-MB-231 and HCC1599 VMTs. **(C)** Gene Ontology Biological Processes pathway analyses for MDA-MB-231 stromal-like tumor population, **(D)** MDA-MB-231 cycling tumor population, and **(E)** MDA-MB-231 tumor population. **(F)** Gene Ontology Biological Processes pathway analyses for HCC1599-1 tumor population and **(G)** HCC1599-2 tumor population.

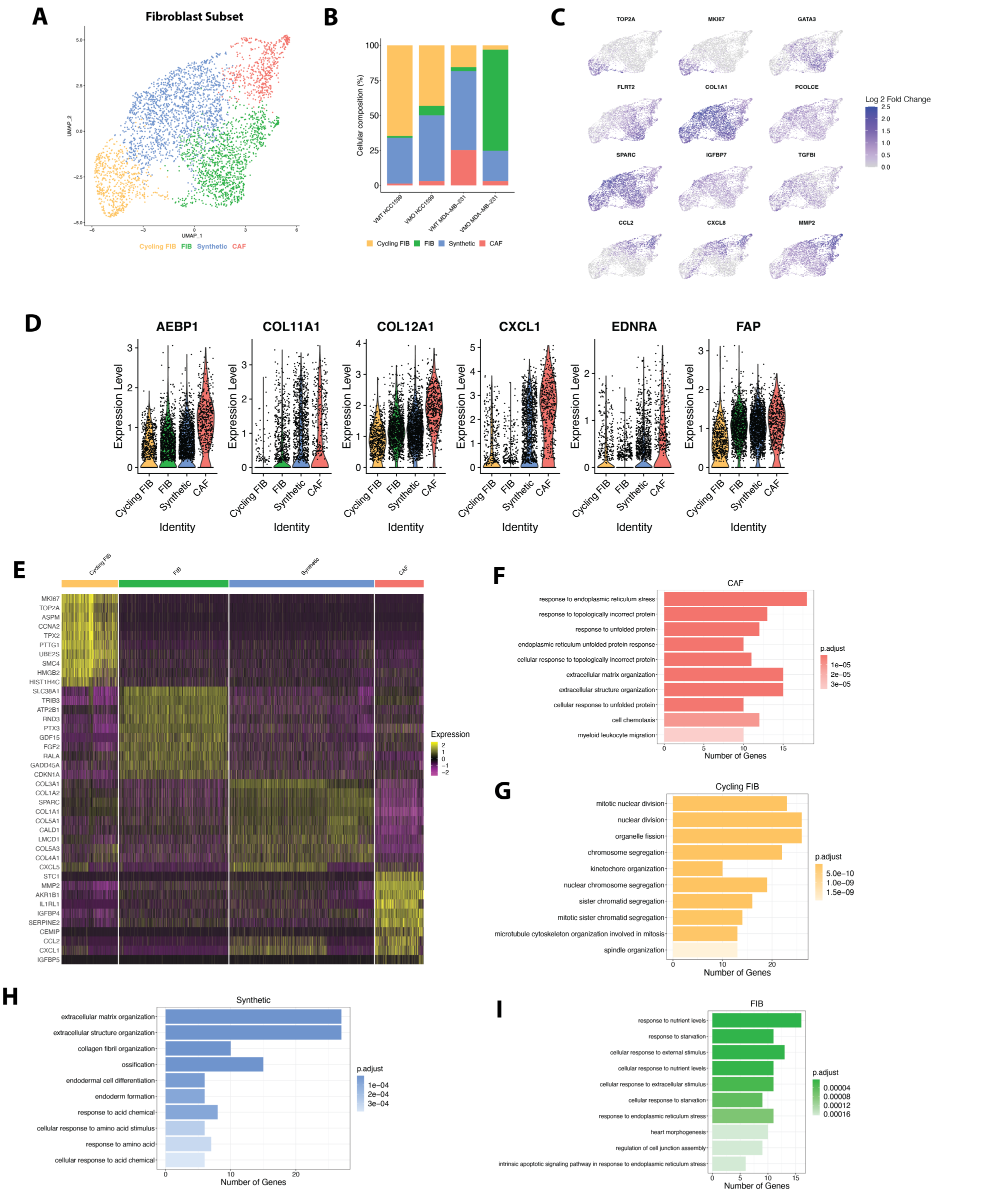

Fig. A3: **Fibroblast subset from fully integrated dataset shows increase in CAF cell signature in MDA-MB-231 VMT.** **(A)** Unsupervised clustering of integrated fibroblast dataset reveals four distinct clusters with **(B)** various proportions of each dataset in the cluster. **(C)** Expression profiles of differentially expressed genes (DEGs). **(D)** Violin plots showing differentially expressed CAF markers across fibroblast types. **(E)** Heatmap of top 10 differentially expressed genes for each cluster. **(F)** Pathway analysis for the top 100 DEGs for each cluster for the CAFs, **(G)** cycling fibroblasts, **(H)** synthetic fibroblasts, and **(I)** fibroblasts.

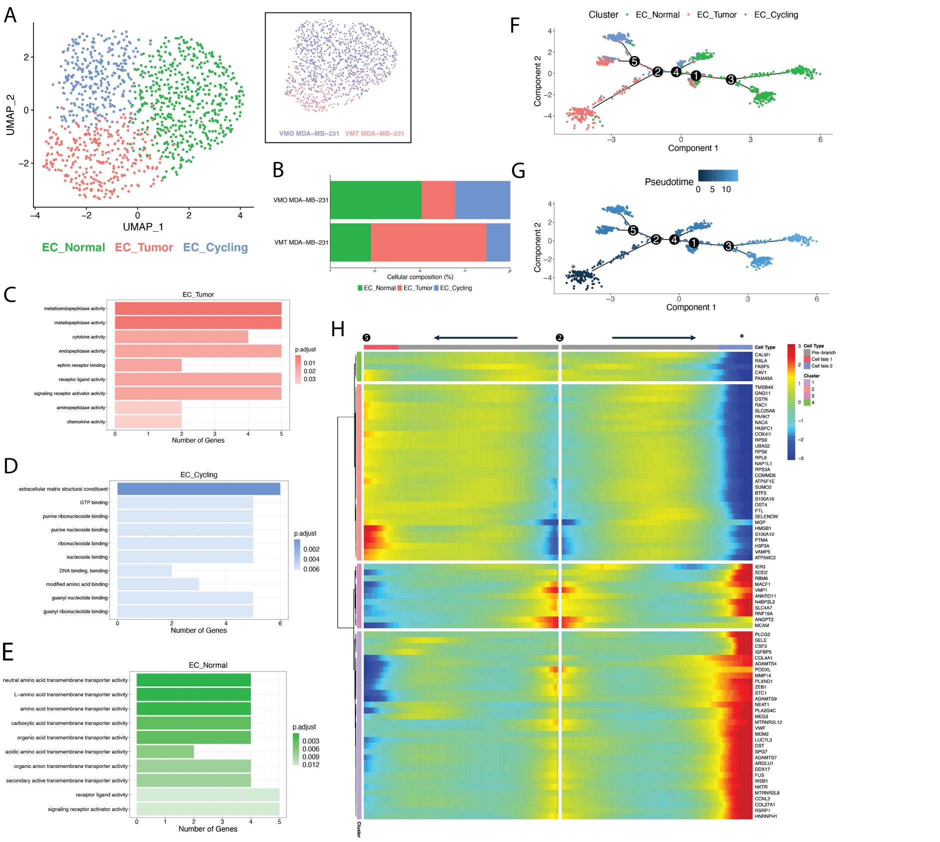

Fig. A4: **EC subset pseudotime analysis reveals enrichment of tumor-associated EC signature in MDA-MB-231 VMT.**

(A) Integration and unsupervised clustering of the endothelial cells (ECs) from the VMO and VMT MDA-MB-231 reveal three populations. Subset: the distribution of cells based on dataset in the dimensional space. **(B)** The proportion of each cluster from each dataset. Note the distinction between the normal and tumor populations. **(C)** Pathway analysis on the top 30 differentially expressed genes for each cluster shows the normal EC with an increase in receptor-ligand activity and transmembrane transporter activity, **(D)** the tumor EC have an increase in metalloendopeptidase activity and cytokine activity, and **(E)** the cycling EC have an increase in DNA binding. **(F)** Pseudotime on the MDA-MB-231 ECs supports the prior clustering and reveals a branch off branch point 2 that is mainly tumor associated (denoted with *). **(G)** Assigning pseudotime emphasizes differences between branch points and shows a starting time with normal EC transitioning into tumor EC. **(H)** The heatmap displays the expression of genes associated with specific branches. Branch 2 is positioned at the center of the heatmap, serving as the midline. Moving towards the left from the center indicates gene expression linked to cells at branch point 5. Moving towards the right indicates gene expression associated with tumor ECs.

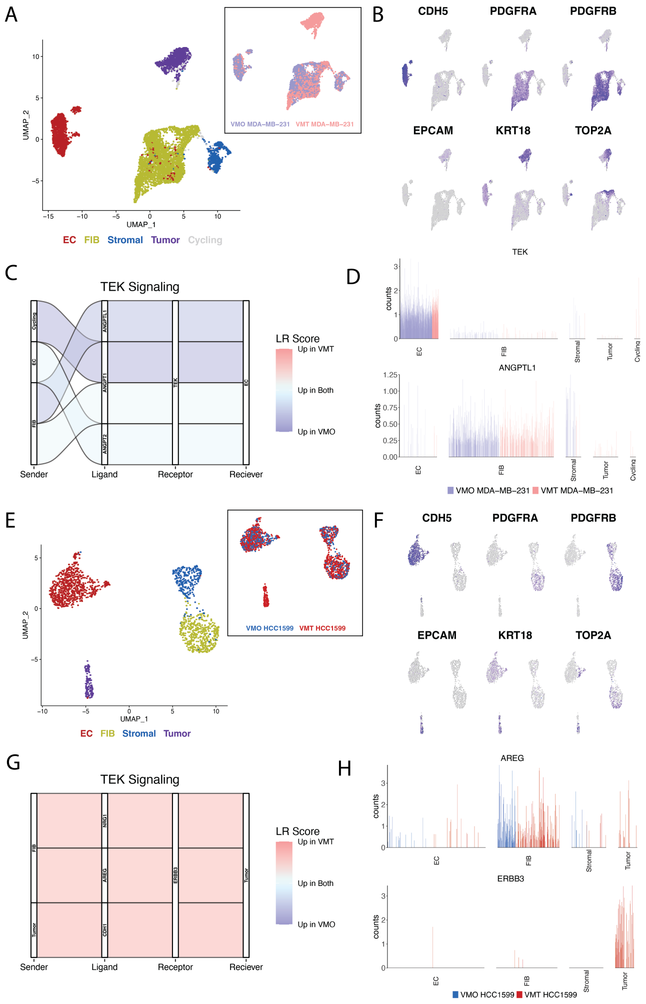

Fig. A5: **Tumor-stromal drug targets are identified in VMO to VMT integrated datasets.** **(A)** Integration with the VMO MDA-MB-231 and VMT MDA-MB-231 datasets showing five cell types of cycling cells, ECs, fibroblasts (FIB), stromal cells (Peri), and tumor **(B)** as demonstrated by the expression of key marker genes. **(C)** Angiopoietin-1 receptor (TEK) cell-cell communication comparing the strength of the signaling between the VMO and VMT. Sender = cell type that produces the ligand, Receiver = cell type that produces the receptor. **(D)** Expression of TEK ligand angiopoietin like 1 (ANGPTL1) and TEK in the integrated dataset. Each bar refers to the expression in a single cell. **(E)** Integration with the VMO HCC1599 and VMT HCC1599 datasets showing four cell types of endothelial cells (ECs), fibroblasts (FIB), pericytes (Peri), and tumor **(F)** as demonstrated by the expression of key marker genes. **(G)** Erb-B2 Receptor Tyrosine Kinase 3 (ERBB3/HER3) cell-cell communication comparing the strength of the signaling between the VMO and VMT. **(H)** Expression of ERBB3 ligand amphiregulin (AREG) and ERBB3 in the integrated dataset.

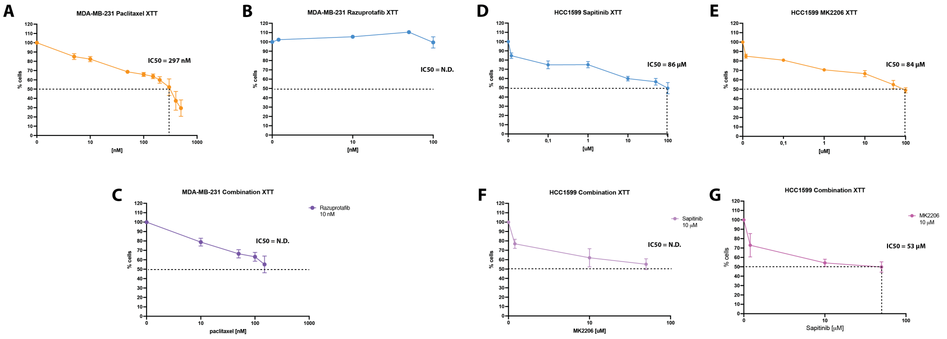

Fig. A6: **Dose response curves for MDA-MB-231 and HCC1599 in monolayer or suspension monocultures.** **(A)** Dose response curve for paclitaxel treatment in MDA-MB-231 2D monocultures. **(B)** Dose response curve for razuprotafib treatment in MDA-MB-231 2D monocultures. **(C)** Dose response curve for combination treatment in MDA-MB-231 2D monocultures. **(D)** Dose response curve for sapitinib treatment in HCC1599 suspension monocultures. **(E)** Dose response curve for MK2206 treatment in HCC1599 suspension monocultures. **(F)** Dose response curve for combination treatment in HCC1599 suspension monocultures. **(G)** Dose response curve for combination treatment in HCC1599 suspension monocultures. N.D. = not determined
